# Single-particle cryo-EM using alignment by classification (ABC): the structure of *Lumbricus terrestris* haemoglobin

**DOI:** 10.1107/S2052252517010922

**Published:** 2017-08-31

**Authors:** Pavel Afanasyev, Charlotte Seer-Linnemayr, Raimond B. G. Ravelli, Rishi Matadeen, Sacha De Carlo, Bart Alewijnse, Rodrigo V. Portugal, Navraj S. Pannu, Michael Schatz, Marin van Heel

**Affiliations:** a Institute of Biology Leiden, Leiden University, 2333 CC Leiden, The Netherlands; b Institute of Nanoscopy, Maastricht University, 6211 LK Maastricht, The Netherlands; c Institute of Molecular Biology, University of Zurich, Switzerland; d Netherlands Centre for Electron Nanoscopy (NeCEN), Einsteinweg 55, 2333 CC Leiden, The Netherlands; e FEI Company/Thermo Fisher Scientific, Eindhoven, The Netherlands; f Brazilian Nanotechnology National Laboratory (LNNANO), Campinas, SP, Brazil; g Leiden Institute of Chemistry, Leiden University, 2333 CC Leiden, The Netherlands; h Image Science Software GmbH, 14193 Berlin, Germany; i Department of Life Sciences, Imperial College London, England

**Keywords:** alignment by classification, angular reconstitution, MSA, worm haemoglobin, cryo-EM

## Abstract

Single-particle cryogenic electron microscopy (cryo-EM) can now yield near-atomic resolution structures of biological complexes. However, the reference-based alignment algorithms commonly used in cryo-EM suffer from reference bias, limiting their applicability (also known as the ‘Einstein from random noise’ problem). Low-dose cryo-EM therefore requires robust and objective approaches to reveal the structural information contained in the extremely noisy data, especially when dealing with small structures. A reference-free pipeline is presented for obtaining near-atomic resolution three-dimensional reconstructions from heterogeneous (‘four-dimensional’) cryo-EM data sets. The methodologies integrated in this pipeline include *a posteriori* camera correction, movie-based full-data-set contrast transfer function determination, movie-alignment algorithms, (Fourier-space) multivariate statistical data compression and unsupervised classification, ‘random-startup’ three-dimensional reconstructions, four-dimensional structural refinements and Fourier shell correlation criteria for evaluating anisotropic resolution. The procedures exclusively use information emerging from the data set itself, without external ‘starting models’. Euler-angle assignments are performed by angular reconstitution rather than by the inherently slower projection-matching approaches. The comprehensive ‘ABC-4D’ pipeline is based on the two-dimensional reference-free ‘alignment by classification’ (ABC) approach, where similar images in similar orientations are grouped by unsupervised classification. Some fundamental differences between X-ray crystallography *versus* single-particle cryo-EM data collection and data processing are discussed. The structure of the giant haemoglobin from *Lumbricus terrestris* at a global resolution of ∼3.8 Å is presented as an example of the use of the ABC-4D procedure.

## Abstract 2

An efficient and fast pipeline is presented for obtaining near-atomic resolution structures from large single-particle cryo-EM data sets. The approach is virtually reference-free and is therefore less prone to the perils of reference bias.

## 1. Introduction   

Since the beginning of single-particle electron microscopy more than three decades ago, we have seen a continuous improvement in electron-microscopy instrumentation, in (cryogenic) specimen-preparation techniques and in image-processing methodologies running on ever more powerful (parallel) computers (van Heel *et al.*, 2000 ▸; Henderson, 1995 ▸; Adrian *et al.*, 1984 ▸; van Heel & Frank, 1981 ▸). The advent of digital image detectors and automatic data collection has greatly facilitated the systematic collection of large data sets without human interaction (Suloway *et al.*, 2005 ▸). These developments, together with the impressive recent advances in ‘direct’ electron detectors (McMullan *et al.*, 2009 ▸; Milazzo *et al.*, 2005 ▸; Faruqi *et al.*, 2005 ▸), have led to the solution of biological structures with near-atomic resolution (for recent reviews, see Cheng, 2015 ▸; Cheng *et al.*, 2015 ▸; Kühlbrandt, 2014 ▸; Nogales, 2016 ▸). The new direct electron detectors represent a significant increase in detective quantum efficiency (DQE) for high-resolution data collection (McMullan *et al.*, 2014 ▸).

Fast movie-mode data acquisition now allows one to routinely compensate for specimen drift during exposure, reviving an important ‘old’ idea (Kunath *et al.*, 1984 ▸). With the large field of view of modern cameras one can now also correct for local beam-induced movements around individual particles (Campbell *et al.*, 2012 ▸; Brilot *et al.*, 2012 ▸; Li *et al.*, 2013 ▸). The collection of large data sets is essential to obtain high-resolution results by bringing even subtle differences between the images to statistical significance. For example, the statistical properties of all individual pixels in a detector can be characterized from a large data set and can subsequently be used to correct for imperfection of the camera (Afanasyev *et al.*, 2015 ▸).

Obtaining near-atomic resolution structures is, however, still challenging and various pitfalls must be avoided. Three-dimensional reconstructions result from the classification and averaging of a large number of low-dose, low signal-to-noise ratio (SNR) images of particles in random orientations. A major pitfall of such single-particle cryo-EM processing is ‘reference bias’, which results from correlation alignment of noisy images with respect to specific references (Boekema *et al.*, 1986 ▸; Dube *et al.*, 1993 ▸; Stewart & Grigorieff, 2004 ▸). It is essential to avoid reference bias as much as possible both in detecting particles in the micrographs or in determining their spatial orientations (van Heel, 2013 ▸; Henderson, 2013 ▸; Subramaniam, 2013 ▸). One important source of reference bias can be avoided by exploiting only information that emerges from the data set itself, without the use of external references (‘starting models’). If the data set is good enough to yield atomic resolution results, then this information is intrinsically present in the data set and all one needs is a systematic approach to extract this information, rather than needing an external starting model.

Our primary tool for extracting the inherent data-set information is multivariate statistical analysis (MSA): eigenvector–eigenvalue data compression and automatic unsupervised classification (van Heel *et al.*, 2009 ▸, 2016 ▸). The linear MSA approaches [members of the principal component analysis (PCA) family] have proven to be very powerful tools in single-particle cryo-EM, for example for sorting structural heterogeneities in cryo-EM samples. Structural heterogeneity of the sample is one of the most rewarding challenges of single-particle cryo-EM, allowing one to visualize three-dimensional molecules in action (in ‘four dimensions’; Klaholz *et al.*, 2004 ▸; Zhou *et al.*, 2015 ▸; Fischer *et al.*, 2010 ▸; Cianfrocco *et al.*, 2013 ▸). MSA is used here especially for a four-dimensional extension of the reference-free ‘alignment-by-classification’ (ABC) approach (Dube *et al.*, 1993 ▸).

We demonstrate our approach using the giant haemoglobin from *Lumbricus terrestris* as a test sample. This molecule, also known as erythrocruorin, has been a favourite test sample in electron microscopy because of its large size (3.6 MDa) and its ease of specimen preparation (Crewe, 1983 ▸; Boekema & van Heel, 1988 ▸; Schatz *et al.*, 1995 ▸; de Haas *et al.*, 1996 ▸; Mouche *et al.*, 2001 ▸; van Bruggen & Weber, 1974 ▸). Oxygen-carrying haemoglobins are present in the red blood cells of most vertebrates as ∼67 kDa tetramers (Perutz, 1978 ▸). In some invertebrates, including the earthworm *L. terrestris*, haemoglobins are large extracellular oligomers with *D*6 point-group symmetry comprised of 144 haem-containing subunits.

Giant haemoglobins are arranged as 12 protomers (1/12th units) assembled around a central scaffold of 36 linker chains (Royer *et al.*, 2000 ▸). Each protomer contains 12 haem groups and possesses a local threefold symmetry axis (which was first revealed by cryo-EM; Schatz *et al.*, 1995 ▸) which relates three *abcd* tetramers to each other (Royer *et al.*, 2000 ▸). The *abcd* tetramers themselves have a quasi-*C*2 symmetry axis, whereby the *ad* and *bc* dimers each share a further local dyad axis that is not perpendicular to the quasi-*C*2 axis of the tetramer. A number of crystallographic studies of the giant annelid haemoglobins are currently available: that from *L. terrestris* at 5.5 Å resolution (Royer *et al.*, 2000 ▸) and at 3.5 Å resolution (Royer *et al.*, 2006 ▸), and that from *Glossoscolex paulistusa* at 3.2 Å resolution (Ruggiero Bachega *et al.*, 2015 ▸).

Since the first cryo-EM worm-haemoglobin structure was obtained at 30 Å resolution (Schatz *et al.*, 1995 ▸), single-particle cryo-EM has improved significantly. This is especially true with regard to the computing power that is available today, allowing data sets that are orders of magnitude larger to be processed, yielding orders of magnitude more information. A recent cryo-EM worm-haemoglobin data set acquired on a FEI Falcon I camera resulted in a 6 Å resolution map that was deposited in the EMDB (EMD-2825). In the present study, the data were acquired in movie mode on a Falcon II camera, resulting in a 3.8 Å resolution map (EMD-3434). In our new robust processing pipeline for large data sets we have incorporated various recent developments in the *IMAGIC*-4*D* software system (van Heel *et al.*, 1996 ▸, 2012 ▸), which are detailed below in the step-by-step procedures.

## 2. Methods and results   

**Figure 1:**
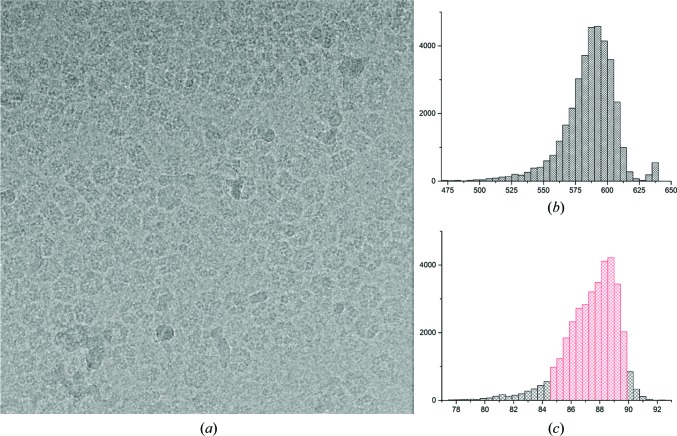
Full data set statistics. (*a*) Typical micrograph (the average of a seven-frame movie; coarsened to a size of 512 × 512) of the diluted worm-haemoglobin haemolymph. The images contain concentrated particles as well as some ‘junk’. Histograms of the average density (*b*) and the standard deviation (*c*) of all of the frames in the data set (36 645 frames or a total of 5235 movies). Images falling within the standard-deviation range marked in red were used for characterization of the camera properties.

The quality of the final cryo-EM results depends on many aspects of the recorded data even prior to any data processing. These aspects include the purity and stability of the sample (Kastner *et al.*, 2008 ▸), fluctuations in preparing vitrified samples, the cryo-EM instrumentation used and how this instrumentation was used *etc.* Each single-particle cryo-EM data set thus has its own unique characteristics and must be processed accordingly. The data-processing pipeline used for cryo-EM data analysis therefore must be flexible and provide a large variety of computational tools. Once certain procedures have proven successful for a specific task, such procedures can be grouped to facilitate repeated or iterative processing. The logistics of the processing requires that each image, or each movie frame, is associated with its own ‘metadata’ such as standard deviation, average density, defocus parameters, Euler angle orientations *etc.* The *IMAGIC* system was designed around the stack philosophy, where many individual images can be stored in a single file, with each image having its own metadata header. Mechanisms are in place to automatically inherit or take over metadata and to sort, activate or deactivate individual images for processing based on all possible parameters. Most decision making is based on purely statistical measures (histograms of the header metadata), rather than on the visual assessment of individual micrographs or molecular images (see, for example, Fig. 1 ▸). At the end of each of the general processing steps in the pipeline discussed below, the relevant commands are listed.

### 2.1. Sample preparation and data collection   

A few droplets of ‘pure’ haemolymph were extracted from the *L. terrestris* heart (the seventh segment from the head) and then diluted 30-fold with buffer prior to freeze-plunging. The resulting images have a high particle concentration and contain some extraneous material (Fig. 1 ▸). The procedures discussed below were well capable of *in silico* purification of the ‘dirty’ sample.

The images were collected on an FEI Titan Krios microscope as detailed in Appendix *A* [App appa]. In cryo-EM, when the micrographs are recorded close to focus (less than ∼0.5 µm underfocus), the complexes can often hardly be seen owing to the lack of phase contrast in the low-frequency regime. In far-from-focus images, on the other hand (more than ∼2 µm underfocus), the high-frequency information becomes compromised. Given the good contrast/visibility of the particles, we targeted the data collection at underfocus values of 1.0 and 1.2 µm. The experimental defocus spread, in combination with some astigmatism, then yielded a good homogeneous coverage of the image information over all spatial frequencies (van Heel *et al.*, 2000 ▸).

### 2.2. Data conversion and initial assessment of the micrographs   

The images collected by the FEI *EPU* data-acquisition program, in an FEI–MRC format, were converted to *IMAGIC* stacks using the *em*2*em* conversion program (http://www.imagescience.de/em2em). This conversion provides each individual image (movie frame) with its own metadata header (available throughout further processing). The total data set of 5235 movies contained a significant number of bad images (grid-bar edges, strong ice contamination *etc.*). These were excluded from the data set based on simple averages and standard deviations (Fig. 1 ▸). The final ‘raw’ data set thus contained 4062 good movies (78% of the full data set; a total of 28 434 movie frames).

Relevant commands: EM2EM, SURVEY-DENSITIES, DISPLAY, HEADERS, SELECT-IMAGES, EXCLUSIVE-COPY.

### 2.3. Camera correction and data pre-processing   

An *a posteriori* camera correction is then applied to minimize spurious correlations owing to camera imperfections (Afanasyev *et al.*, 2015 ▸). Large data sets collected with the same camera reflect the characteristics of each individual pixel. The camera correction is performed after removing extreme outlier images from the data set, but prior to movie alignments. Fig. 1 ▸(*c*) shows the histogram of standard deviations of all frame images; areas marked in red correspond to the frames selected for calculating the camera normalization statistics. The normalization is then applied to the full data set, resulting in the basic data set for all further processing. Different types of filtering are applied to the data set for different purposes. To find the contrast transfer function (CTF) defocus parameters, the low-frequency components (less than ∼0.05 or 0.1 of the Nyquist frequency) are best taken out altogether to avoid wrap-around artefacts leading to ‘crosses’ in the spectra. At the same time, however, after CTF correction (see §[Sec sec2.6]2.6) we need significant levels of low-frequency information to aid in particle picking. We then nevertheless still need to remove the very low-frequency information (less than ∼0.02 of the Nyquist frequency) to suppress gradual background fluctuations that are not associated with the actual particle information. At this stage of processing the contrast of underfocus cryo-EM images is inverted to make proteins ‘white’ against a dark background for processing (standard convention).

Relevant commands: CAMERA-NORM, HEADERS, SELECT-IMAGES, EXCLUSIVE-COPY, SET-INACTIVE, PREPARE-IMAGES, CUT-IMAGE, FILTER-IMAGE, IMAGE-MENU.

### 2.4. Correction of anisotropic magnification   

**Figure 2:**
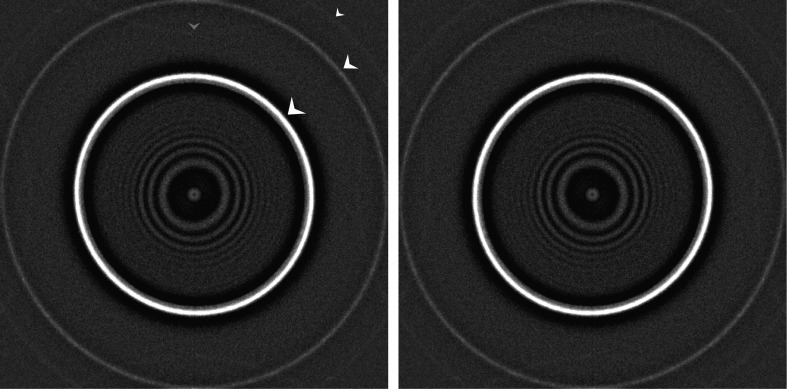
Anisotropic magnification of the worm-haemoglobin data set became evident from an eigenvector analysis of all amplitude spectra (see §[Sec sec2.5]2.5). The second eigenimage revealed a 2.6% ellipticity of the ∼3.6 Å water ring (marked with large arrow in the left spectrum) and the 2.2 Å water ring (medium-sized arrow). A further water/ice ring can be seen at 1.8 Å (small arrow), which is only visible in the corners of the image (beyond the Nyquist frequency) but reflects back into the image everywhere else owing to aliasing (grey arrow). The right spectrum is the same as the left spectrum but is mirrored horizontally, thus changing the ellipticity direction. The ellipticity is in a close-to-diagonal (∼36°) direction such that the vertical and horizontal magnifications in the data set are almost identical. The Thon rings visible in the centre are elliptical owing to an astigmatism of ∼1000 Å. This astigmatism ellipticity is unrelated to the ellipticity of the water rings. The two effects may become entangled if the anisotropic magnification is not corrected prior to CTF determination.

We noticed from the MSA processing of all spectra (§[Sec sec2.5]2.5) that our FEI Titan Krios microscope, equipped with a *C*_S_ corrector (http://www.ceos-gmbh.de/English/products/cs.html), here yielded images with an anisotropic magnification difference of 2.6%, where the highest magnification was in a 36.2° diagonal direction. For our 300 Å diameter particle (sampled at ∼1.12 Å per pixel) this implies an orientation-dependent magnification fluctuation of ∼4 pixels at the outer edge of the structure, seriously limiting the achievable resolution (to ∼5.5 Å). This anisotropy was detected in the data set itself as an ellipticity of the ∼3.6 and ∼2.2 Å water rings in the MSA-based spectrum analysis (Fig. 2 ▸). A routine was implemented to re-interpolate the raw data set (see §[Sec sec3]3). This anisotropic magnification compensation changed the average pixel size from ∼1.12 to ∼1.11 Å, because the images were magnified to the maximum magnification found in the raw data (demagnification would cause sharp edges to enter at the border).

Relevant commands: MSA-RUN, FIND-ANISOTROPIC-MAGNIFICATION, ANISOTROPIC-MAGNIFY.

### 2.5. Determining the CTF parameters   

**Figure 3:**
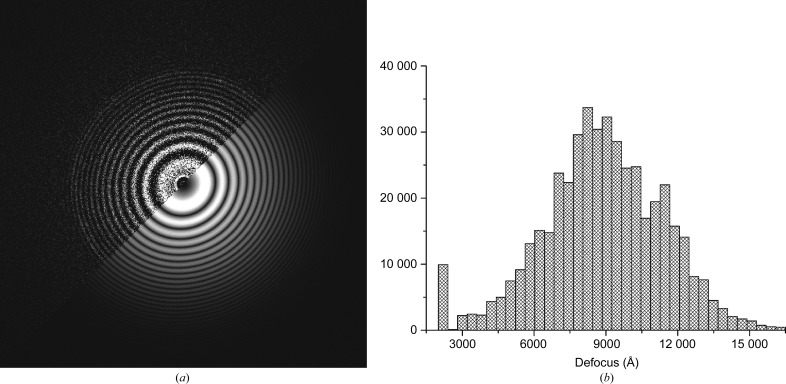
(*a*) Selected class average of individual spectra with fitted theoretical spectrum. Top left, experimental class-average spectrum (1280 × 1280 patches). Bottom right, theoretical CTF amplitudes at a defocus of 7913/9047 Å (astigmatism of 1134 Å at an angle of 133°). Thon rings in good spectra are visible to beyond ∼2/3 of the Nyquist frequency, corresponding to a resolution of ∼3.1 Å. (*b*) Histogram of the defocus values of ∼450 000 movie-frame patches. The peak at the low-defocus edge of this histogram corresponds to outliers with a defocus less than 0.22 µm (the search limit), which were ignored.

The electron microscope is a phase-contrast microscope. The linear transfer of the image information in this instrument is described by the phase contrast transfer function (CTF), which starts at zero at the Fourier-space origin and oscillates around zero as function of spatial frequency (Scherzer, 1949 ▸; van Heel, 1978 ▸; van Heel *et al.*, 2000 ▸). Our automatic defocus and astigmatism determination is based on finding the best correlation between theoretical spectra and experimentally measured spectra (Mindell & Grigorieff, 2003 ▸; van Heel *et al.*, 2000 ▸). Here, we used the average of the power spectra of individual movie frames from a single movie as the basic measurement for CTF determination. These individual movie-frame spectra are shift-invariant, implying that we can determine the CTF prior to movie-frame alignments. The average spectra are filtered to suppress the influence of background ramps (Mindell & Grigorieff, 2003 ▸; van Heel *et al.*, 2000 ▸). In line with the ‘full-data-set CTF correction’ strategy (van Heel *et al.*, 2012 ▸), the large set of spectra is then submitted to eigenvector analysis and automatic unsupervised classification (Sander *et al.*, 2003 ▸; Louys *et al.*, 1989 ▸; van Heel *et al.*, 2000 ▸, 2012 ▸). The resulting class averages are then used for the actual CTF determination (Fig. 3 ▸). Here, the spectra were calculated based on 16 overlapping ‘checkerboard’ patches (1280 × 1280 pixels) taken from the 4096 × 4096 movie frames.

Relevant commands: CUT-IMAGES, CREATE-PRETREATED-AMPLITUDES, MOVIE-SPECTRUM, MSA-RUN, MSA-CLASSIFY, MSA-SUM, EIGEN-FILTERING, CTF-FIND.

### 2.6. CTF correction   

Each individual patch image is assigned the defocus parameters of its class. Members of ‘poor’ quality classes are excluded from further processing. CTF correction is performed by phase-flipping individual movie frames (or 1280 × 1280 patches) at full resolution, thus maintaining the original power-spectrum distribution of the data. Because of experimental defocus variations throughout the data set (Fig. 3 ▸ *b*) and the presence of astigmatism, this strategy leads to a smooth distribution of both signal and noise in Fourier space (van Heel *et al.*, 2000 ▸). CTF correction in the early phases of the processing largely concentrates the information from a particle, spread out by the point-spread function, back to within the boundaries of that particle (see Fig. 2 of van Heel *et al.*, 2000 ▸). Thus, one can avoid maintaining large areas around the individual particles during processing. Moreover, an early CTF correction makes the molecular images comparable throughout processing.

Relevant commands: HEADERS, CTF-FLIP.

### 2.7. Movie alignments   

**Figure 4:**
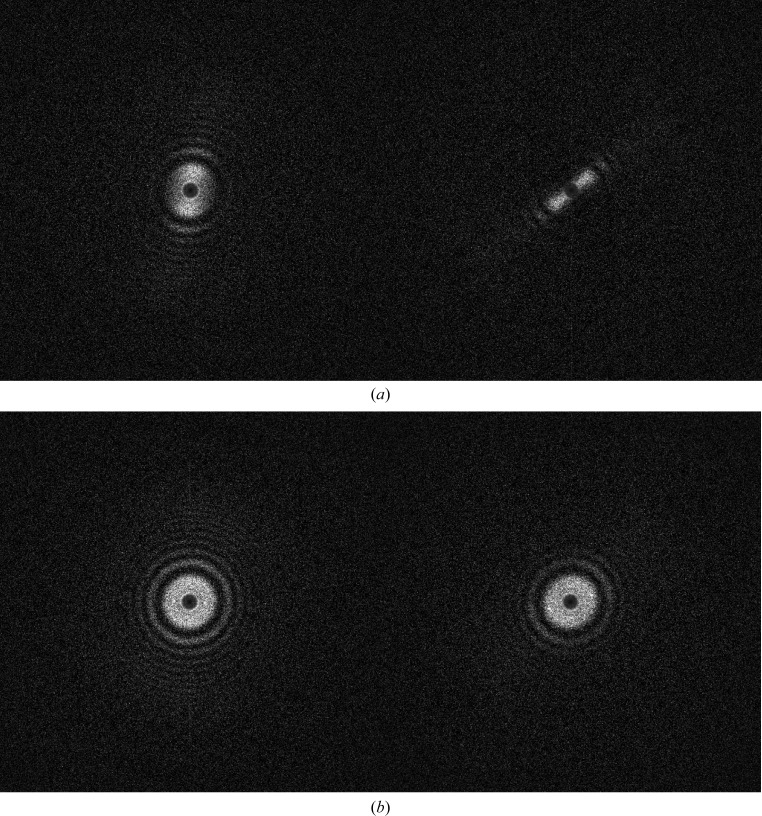
Two spectra of drifted movies: (*a*) before and (*b*) after movie alignments. The second movie in (*a*) experienced a stronger drift, impeding the restoration of the high-resolution information (only two ‘zeroes’ in the direction of the drift were restored).

During the exposure time of a micrograph, the sample may suffer drifts, which smear out the high-resolution information in the drift direction. These drifts can be corrected by movie alignments (Kunath *et al.*, 1984 ▸; Campbell *et al.*, 2012 ▸; Ripstein & Rubinstein, 2016 ▸). Movie alignments are hampered by high-frequency fixed-pattern noise. This makes it necessary to apply extreme low-pass filters prior to movie alignments. An *a posteriori* camera correction (§[Sec sec2.3]2.3) significantly minimizes spurious correlations from such fixed background patterns and thus improves movie alignments (Afanasyev *et al.*, 2015 ▸). We perform translational movie alignments on the full movie frames or, as was performed here, on extracted patch movies (1280 × 1280). The algorithm used is a conventional cross-correlation alignment of each frame with respect to a running average of the (aligned) frames (Kunath *et al.*, 1984 ▸; Campbell *et al.*, 2012 ▸). Figs. 4 ▸(*a*) and 4 ▸(*b*) illustrate ‘bad’ spectra before and after alignment, showing that even seriously drifted movies can be restored. However, when extreme drifts occur within the integration time of a single frame, the aligned movie will lack high resolution in the drift direction (Fig. 4 ▸ *a*, right) and should be discarded.

Relevant commands: COARSEN-IMAGES, ALIGN-MOVIE-FRAMES, SUM-IMAGES, MOVIE-SPECTRUM, SELECT-IMAGES, MANIPULATE-PLT-FILE, HEADERS.

### 2.8. Initial particle picking   

**Figure 5:**
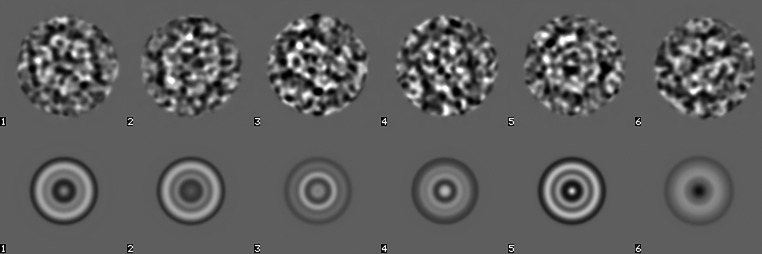
Six manually selected particles (top row) used as templates for an initial competitive particle picking and their rotational averages (bottom row).

For an initial particle picking, the CTF-corrected and aligned movie sums are coarsened and band-pass filtered. As an alternative to the preferred method of local variance-based particle picking (van Heel, 1982 ▸, 2013 ▸), we here used rotationally averaged particles to create a first set of featureless templates for competitive particle picking from the 500 movie sums with the largest defocus values (Fig. 5 ▸).

This first round of particle picking with indiscriminate references yields ‘dirty’ results. Picking artefacts include crystalline ice, carbon foil edges, fragments of molecules, contamination *etc.* These are largely excluded based on their high standard-deviation values. False-positive picks at the low end of the standard-deviation histograms represent random background fluctuations and may also be excluded. Combinations of selection criteria can also be used (average density, sigma and cross-correlation coefficient *etc.*). Here, ∼20 000 picks from a total of ∼30 000 were deemed to be true haemoglobin particles and the remainder were deemed to be false positives.

Relevant commands: COARSEN-IMAGES, PICK-PARTICLES, CUT-IMAGES, FILTER-IMAGES, MASK-IMAGE, AVERAGE-ROTATIONAL, SURVEY-DENSITIES, HEADERS, SELECT-IMAGES.

### 2.9. Eigenvector data compression and unsupervised classification   

**Figure 6:**
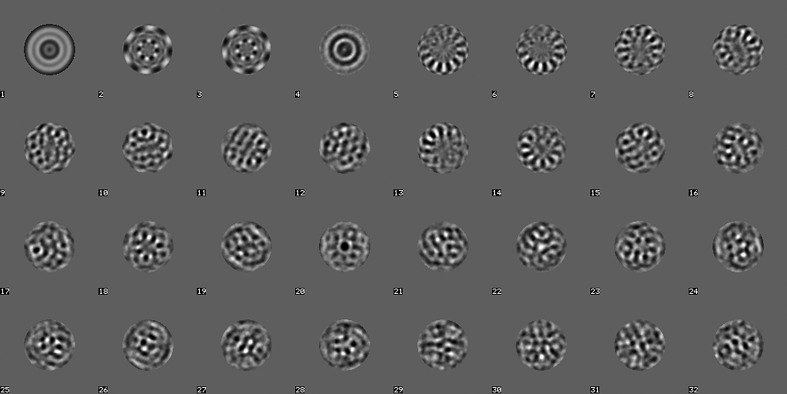
The first 32 eigenimages of 20 000 picked particles. The first eigenimage is rotationally symmetric, reflecting this property of the first set of particle-picking templates. Eigenimages 2 and 3 reflect the predominant sixfold symmetry of the worm-haemoglobin ‘top views’.

**Figure 7:**
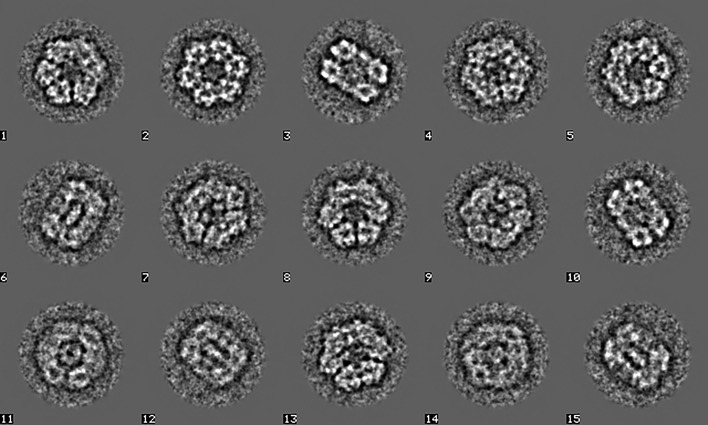
15 class averages of unaligned particles (‘alignment by classification’; ABC) used for an initial ‘random-startup’ three-dimensional reconstruction. Class average 2 corresponds to a top view and class averages 3 and 10 correspond to side views. The other class averages represent various intermediate projection images of the haemoglobin. Since the input images are coarsened (binned) by a factor of four, no information beyond the Nyquist frequency of 1/8.8 Å can be present in the images at this stage.

Whilst simple statistics are very useful for a rough first screening of the picked particles, such screening is insufficient for an in-depth diagnosis of the particle stack. Eigenvector–eigenvalue methods (MSA) are much richer since all orthogonal ‘principal components’ of the data stack are studied simultaneously. MSA with modulation distances (Borland & van Heel, 1990 ▸) was used to analyse the current particle stack (Fig. 6 ▸). Eigenimages 2 and 3 represent the main sixfold-symmetry component of the data set (Dube *et al.*, 1993 ▸). A fast hybrid hierarchical ascendant classification (HAC) strategy is used, which incorporates a random seeds pre-processor within a standard HAC procedure maintaining the principles of ‘minimum added intraclass variance’ (van Heel, 1989 ▸; van Heel *et al.*, 2016 ▸; Ward, 1963 ▸). The worm-haemoglobin class averages (∼20 members per class) were sorted based on class size and overall quality. Some 15 class averages were chosen randomly from the best 500 for an initial Euler-angle assignment (Fig. 7 ▸). These two-dimensional class averages, showing the molecules with a significantly improved signal-to-noise ratio (SNR), were obtained without alignments with respect to specific reference images in an ‘alignment by classification’ (ABC) procedure (Dube *et al.*, 1993 ▸).

Relevant commands: MSA-RUN, MSA-CLASSIFY, MSA-SUM, SORT-IMAGES, HEADERS, SELECT-IMAGES.

### 2.10. Initial three-dimensional reconstruction by ‘random startup’   

**Figure 8:**
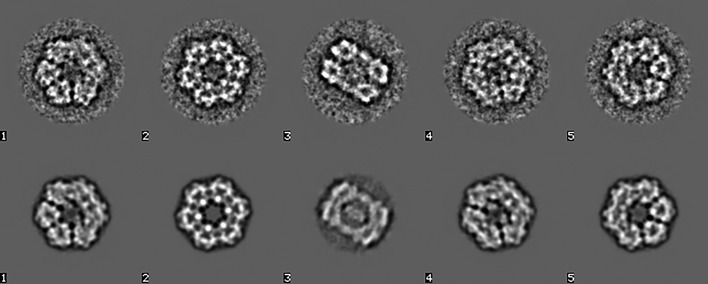
Five class averages used for random startup (top row of Fig. 7 ▸) and the corresponding reprojections from the three-dimensional reconstruction. A good correspondence of the class averages to their reprojections is a necessary condition for a valid reconstruction procedure.

The obtained reference-free class averages are two-dimensional projection images of the underlying three-dimensional structure(s). The conversion from two-dimensional space to three-dimensional space is performed by angular reconstitution (AR; van Heel, 1987 ▸), providing the relative Euler-angle orientations of the *N* input two-dimensional class averages. Random Euler angles are first assigned to each of the class averages. These Euler angles are then refined iteratively by assigning a new orientation to each class average, using the other (*N* − 1) class averages as an anchor set (Schatz *et al.*, 1995 ▸; van Heel *et al.*, 2000 ▸). In our model case of worm haemoglobin, *D*6 symmetry was assumed. The *a priori* assumption made here is that a sufficient number of distinct projections are available. The random-startup procedure can fail when a data set from a (small) low-symmetry complex exhibits strong preferential orientations, implying that the data set is not sufficiently ‘three-dimensional’. After convergence has been achieved, a three-dimensional reconstruction is calculated based on these angles using the weighted back-projection algorithm (Harauz & van Heel, 1986 ▸). The re-projections of the three-dimensional reconstruction can be compared with the corresponding input class averages for validation (Fig. 8 ▸).

A new anchor set is then created from the automatically masked random-startup three-dimensional reconstruction, which is used to determine the orientations of all (500 in our case) class averages. No in-plane alignments, rotational or translational, are used at this stage. A new three-dimensional reconstruction is obtained based on all good class averages. This refinement process (automatic masking → new anchor set → angular reconstitution → three-dimensional reconstruction) is iterated until convergence. The converged map, obtained exclusively from the information present in the data set, is our ‘initial’ three-dimensional reconstruction. Multiple three-dimensional reconstructions (four dimensions) can already be called for to start up highly heterogeneous data sets (van Heel *et al.*, 2012 ▸).

Relevant commands: AUTOMATIC-MASK-IMAGES, ANGULAR-RECONSTITUTION, THREED-RECONSTRUCTION, THREED-FORWARD-PROJECTION, FILTER-IMAGES, THREED-FILTER, THREED-AUTOMATIC-MASKING, SELECT-IMAGES.

### 2.11. Refined particle picking   

**Figure 9:**
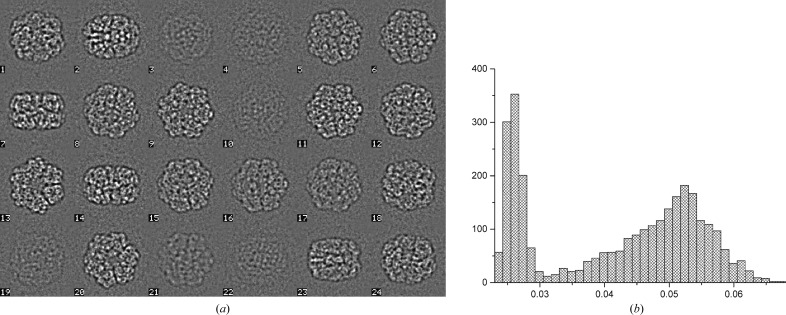
(*a*) Class averages randomly extracted from the total of 3000. High-contrast class averages are associated with good views of the worm haemoglobin, whereas low-contrast class averages are associated with atypical false-positive particle detections. (*b*) The histogram of standard deviations of 3000 class averages (205 764 particles) reveals a bimodal distribution in which virtually all remaining false positives land in low-contrast class averages.

We now use projections from the initial three-dimensional reconstruction(s) as templates for competitive correlation-based particle picking. The already collected low-resolution three-dimensional information leads to a better recognition of the particles in all possible orientations in the micrographs. From the newly picked particles the outliers are again discarded (205 764 of 319 746 were selected). Since we are now searching for all specific views of a complex, the eigenvectors of the picked-particle data set become more dominated by the properties of the actual complex. All remaining false-positive picks lack these characteristics, and their variances are thus not well covered by the eigenvectors. These uncharacteristic images have a poor ‘representation quality’ (the fraction of their variance described by the eigenvectors) with respect to the new set of eigenvectors (van Heel, 1989 ▸). The poor quality of the corresponding class averages is apparent from the resulting bimodal distribution of the class variances (Fig. 9 ▸ *b*). This is thus a very good opportunity to discard all unreliable class averages using their representation quality as the main metric.

Relevant commands: THREED-FILTER, THREED-AUTOMATIC-MASKING, THREED-COARSER-SAMPLING, THREE-FORWARD-PROJECTION, FILTER-IMAGES, PICK-PARTICLES, CUT-IMAGES, HEADERS, SELECT-IMAGES, MANIPULATE-PLT-FILE.

### 2.12. Overall alignment: focus on class averages   

**Figure 10:**
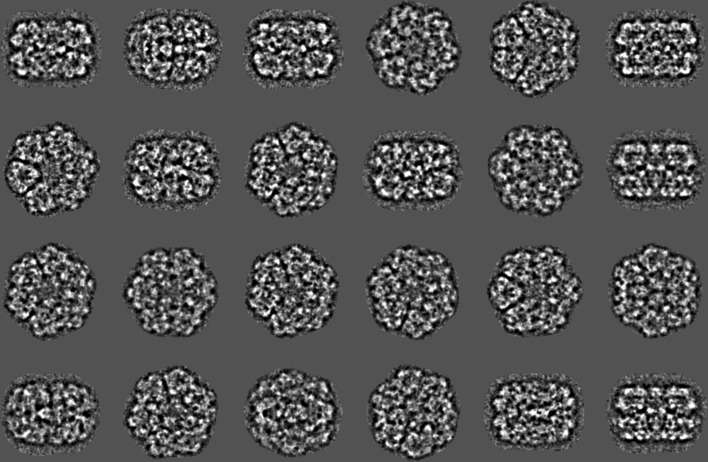
Effect of ABC optimization. After ABC alignment of the full data set, all images are rotated and shifted to a common origin as dictated by the Euler orientations of the class averages. A new MSA classification thus yields a better class average since more molecular images now have a common three-dimensional orientation. Shown here are class averages at an intermediate level of the refinement procedures at a coarsening level of 2 (Nyquist frequency 1/4.4 Å).

With the improved particle picking one finds more particles in all possible orientations. The goal of our iterative ABC procedure is to find the best overall alignment, namely the alignment that maximizes the variance (‘sum of eigenvalues’) in the lower eigenimages (van Heel *et al.*, 2009 ▸, 2012 ▸). We thus first find the Euler orientations (and residual shifts) of the two-dimensional class averages with respect to an anchor set (Schatz *et al.*, 1995 ▸) derived from the three-dimensional reconstruction. These parameters are then applied to all molecular images belonging to this class. This iterative refinement procedure (MSA classification → angle/shift assignment → three-dimensional reconstruction → class-member alignment) brings all molecular images to a common ‘six-dimensional’ coordinate system (‘seven-dimensional’ for heterogeneous data). Similar views become better represented by the main eigenimages of the system, which will increase the chance of them landing in the same class average or in closely related ones (Fig. 10 ▸).

Relevant commands: MSA-RUN, MSA-CLASSIFY, MSA-SUM, SORT-IMAGES, ANGULAR-RECONSTITUTION, THREED-RECONSTRUCTION, THREED-FILTER, THREED-AUTOMATIC-MASKING, THREED-FORWARD-PROJECTIONS, ALIGN-PARALLEL, MOVE-BY-ALIGNED-CLASSUMS, FILTER-IMAGES.

### 2.13. Refinements using MSA in Fourier space   

In the course of the refinement iterations, more high-frequency information materializes and the balance of the high-frequency *versus* low-frequency information becomes more important. At this stage, the MSA unsupervised classification is best performed in Fourier space, restricting the information to a ring mask defining a relevant frequency range. The high-frequency outer edge of this mask is slowly increased from ∼0.4 to ∼0.8 of the Nyquist frequency, gradually including more high-frequency refinements. After each iteration, the resolution can be assessed by Fourier shell correlation (FSC; van Heel & Schatz, 2005 ▸; Harauz & van Heel, 1986 ▸). The class averages in each of the current three-dimensional reconstructions are split into two groups in order to compare their separate three-dimensional reconstructions by FSC. When the FSC threshold exceeds ∼1/2 of the Nyquist frequency this indicates that subsequent refinements should be pursued at a finer (or full) sampling level.

Relevant commands: IMAGE-MENU (HERMITIAN), MSA-RUN, MSA-CLASSIFY, MSA-SUM, FOURIER-SHELL-CORRELATION.

### 2.14. Refinements in four dimensions   

In a broad sense, heterogeneities in the sample are a most fundamental property of cryo-EM data sets. Some heterogeneities are so obvious that they are hardly mentioned, such as the presence of small ice crystals or of 70S ribosome complexes dis­assembling into their 30S and 50S constituents. Such heterogeneities can often be dealt with by simple primary statistics (standard deviations *etc.*). Dealing with subtle conformational differences among otherwise monodisperse complexes, on the other hand, requires software logistics for simultaneously processing multiple structures (Klaholz *et al.*, 2004 ▸; Zhou *et al.*, 2015 ▸). The *IMAGIC* software system has thus been developed to ‘four dimensions’ both in terms of data stacks and data processing (van Heel *et al.*, 2012 ▸).

Here, we use a four-dimensional refinement scheme in which the two-dimensional class averages are randomly assigned to multiple three-dimensional groups, generating a number of independent three-dimensional reconstructions. This stack of three-dimensional reconstructions is subsequently used to create a stack of (three-dimensional/two-dimensional) anchor sets for the next round of refinements. Each two-dimensional class average is reassigned to its ‘best-fit’ three-dimensional group and a new set of reconstructions is computed. The procedure is iterated until convergence is achieved; three-dimensional reconstructions which do not accumulate enough class averages are removed from the four-dimensional pool in a ‘survival of the fittest’ approach.

Our four-dimensional analysis here converged into two different groups, one of which led to a good-quality three-dimensional reconstruction with 56% of the molecular images and the other of which had poorer (FSC) quality and has not been studied further as yet. During the next rounds of ABC Euler-angle assignments, the number of images per class *N* is gradually reduced (even up to *N* = 1). Here, the final Euler angles were assigned to individual molecular images by angular reconstitution. These final images were aligned particle-movie averages (of frames 2–7, excluding the first most-drifted frame 1). The final three-dimensional reconstruction was calculated using frames 2–5 only, excluding frames 6 and 7, which had received the highest accumulated electron dose.

Relevant commands: see §§[Sec sec2.12]2.12 and [Sec sec2.13]2.13.

### 2.15. Quality assessment and anisotropic resolution   

**Figure 11:**
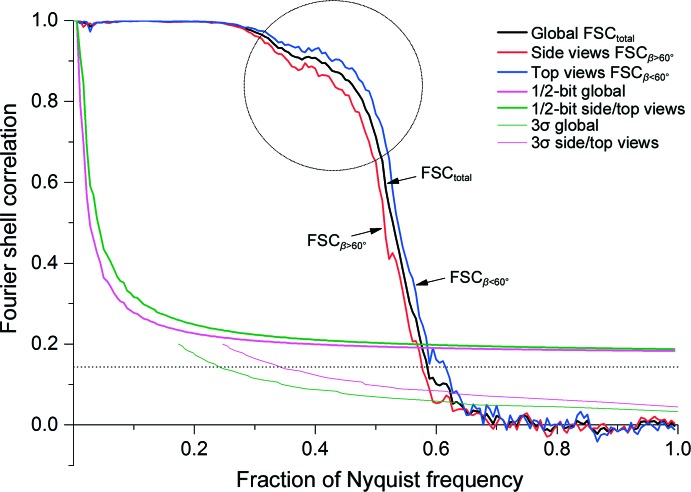
FSC curves of the final three-dimensional reconstruction. (0.6 of the Nyquist frequency corresponds to 1/3.7 Å.) FSC_β<60°_ is based on the preferred top-view class averages (0 < β < 60°); FSC_β>60°_ assesses the contributions of the side-view class averages (60 < β < 90°). The circle indicates the area in which a high FSC value is a good indicator of the quality of the reconstruction.

The FSC of our main final three-dimensional reconstruction at the 1/2-bit threshold indicates that sufficient information was collected for interpretation at ∼3.8 Å resolution (Fig. 11 ▸). The 3σ threshold is below 2/3 of the Nyquist frequency, confirming that a sufficiently fine sampling was used (Orlova *et al.*, 1997 ▸). This is important since the computational procedures (*i.e.* interpolations) can introduce spurious similarities between the compared half-maps close to the Nyquist frequency. Moreover, the FSCs beyond 2/3 of the Nyquist frequency oscillate around zero and remain below 3σ, as expected for random correlations (van Heel & Schatz, 2005 ▸). No obvious high-frequency artefacts were thus introduced. We also include the popular 0.143 threshold (Fig. 11 ▸, dotted line; Rosenthal & Henderson, 2003 ▸), which only has asymptotical validity (huge volumes, high resolution) since radial and symmetry dependencies are neglected (van Heel & Schatz, 2005 ▸). Single-valued resolution criteria are often less relevant than the actual shape of an FSC curve. For example, the relative height of the FSC ‘knee’ area (Fig. 11 ▸) is a good-quality indicator of the map.

Our three-dimensional reconstruction exhibits anisotropic resolution associated with preferential particle orientations. A total of 85 000 molecular images were used for our final three-dimensional reconstruction, of which ∼2/3 were top views and ∼1/3 were side views. The preferred top views make the FSC contributions in this direction stronger than in side-view directions. This effect disappears with the uniform integration over spherical Fourier shells (Harauz & van Heel, 1986 ▸). To quantify this anisotropy, we separately calculate FSC_β<60°_ and FSC_β>60°_, where β is the closest angular distance from the preferred orientation [for C_*n*_ or *D*_*n*_ oligomers (*n* ≥ 3) this orientation may coincide with the major symmetry axis]. The choice of β is flexible, but for β = 60° the number of voxels within a Fourier space sphere in the range 0 ≤ β ≤ 60° equals the number of voxels in the range 60 ≤ β ≤ 90°, making FSC_β<60°_ and FSC_β>60°_ directly comparable in terms of applicable thresholds. The global resolution of our final three-dimensional reconstruction is ∼3.8 Å; the top-view resolution is better (∼3.6 Å), whereas the side-view resolution is restricted to ∼4.0 Å (Fig. 11 ▸).

Relevant commands: THREED-FILTER, THREED-AUTOMATIC-MASKING, FOURIER-SHELL-CORRELATION, HEADERS, SORT-IMAGES.

### 2.16. Data interpretation   

The interpretation of the data is best performed on high-pass filtered data, improving the visibility of the fine details (van Heel, 2000 ▸; Rosenthal & Henderson, 2003 ▸). Any normal filtering of the (half-)maps will affect the numerator and the denominator of the FSC equation (Harauz & van Heel, 1986 ▸) equally and will thus not affect the FSC curve or an FSC-derived resolution criterion. The same is true for the FSC between the map and the atomic model (van Heel *et al.*, 2000 ▸). Filtering, however, can strongly affect the appearance of the maps and their interpretation: ‘…the one and only thing one can do wrong is to interpret the map incorrectly’ (van Heel, 2000 ▸), ‘Although sharpening is a recommended procedure, which usually has a dramatic effect on map interpretability, selecting the ‘correct’ sharpening parameter may be a tricky problem’ (Murshudov, 2016 ▸). Just like the human observer may be fooled by the appearance of the map when manually fitting atomic coordinates (van Heel, 2000 ▸), any automatic coordinate-fitting program may lead to erroneous results if it does not take into account the different properties of the cryo-EM map and the coordinate map (that is, the spectral power distribution, the positivity of the coordinates *versus* the zero average density of phase-contrast cryo-EM results *etc.*). We found it advantageous to normalize the rotationally averaged amplitude spectrum of the cryo-EM map to a unit value (or to the amplitude spectrum of a similar X-ray structure), followed by a (double) Gaussian band-pass filter where the high-frequency 1/*e* value is closely associated with the 1/2-bit FSC threshold (van Heel & Schatz, 2005 ▸). We name this filter the NAS (normalized amplitude spectrum) filter.

**Figure 12:**
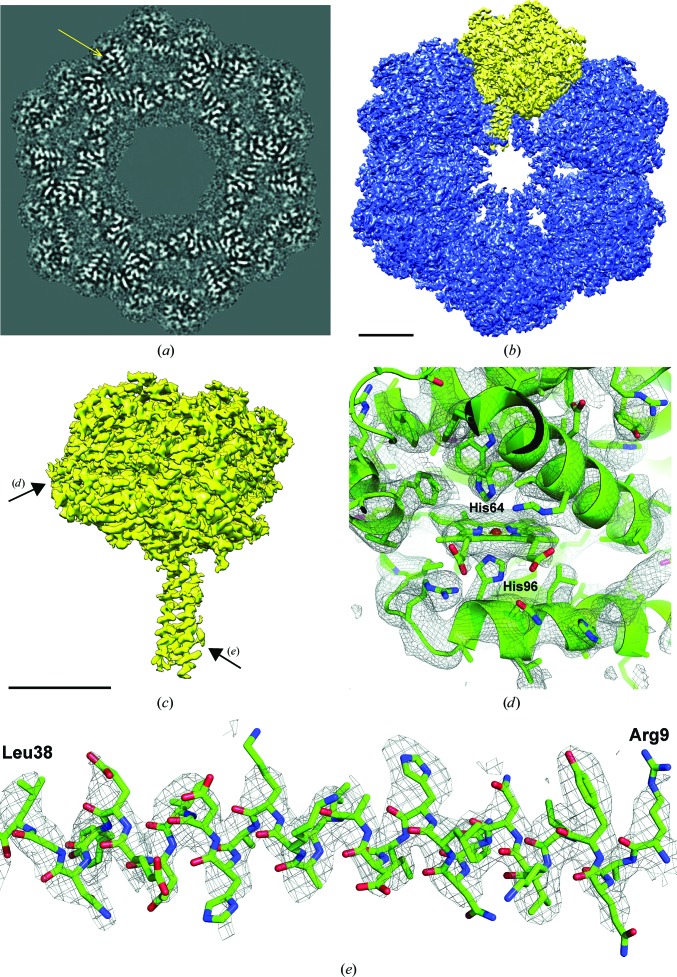
Final cryo-EM map of worm haemoglobin (scale bars indicate 50 Å). (*a*) Slice through the cryo-EM three-dimensional reconstruction perpendicular to the main sixfold symmetry axis, with the yellow arrow pointing at a haem group sandwiched between two α-helices; the proximal histidine contacting the iron in the haem group is visible. (*b*) Surface rendering of the top view (along the sixfold axis); the asymmetric unit (1/12th of the *D*6 reconstruction; see Supplementary Movie S1) is marked in yellow. (*c*) The asymmetric unit (‘protomer’ or ‘1/12th unit’) with arrows pointing at the globin fold shown in (*d*) and the α-helix shown in (*e*). (*d*) View of one the better resolved haem groups: both the proximal and the distal histidine (*b* globin chain: His96 and His64) are well embedded in density. (*e*) View of one α-helix (L_1_ linker chain; Arg9–Leu38) in the triple coiled-coil region: the side chains fit nicely into the three-dimensional reconstruction. The atomic model was deposited as PDB entry 5m3l.

The 12 worm-haemoglobin protomers (Fig. 12 ▸ *c*), each containing 12 haem groups, are clearly discernible in the final three-dimensional reconstruction. The 12 globin folds within each protomer were determined independently (no local symmetry was exploited). The ‘stem’ of the mushroom-shaped 1/12th unit is a heterotrimer of linker chains forming a triple coiled-coil structure. With a global resolution of 3.8 Å, the best areas of the map have a significantly better resolution. We clearly resolved the haem groups (Fig. 12 ▸ *d*) and could distinguish various side chains, especially in the coiled-coil region (Fig. 12 ▸ *e*).

Relevant commands: THREED-FILTER, THREED-AUTOMATIC-MASKING, THREED-SUM-VOLUMES, THREED-RESIZE-VOLUME, THREED-CUT-IMAGE, THREED-SUBTRACT-VOLUMES, THREED-ALIGN-VOLUMES, EM2EM.

## 3. Discussion   

Excellent huge data sets and a coherent data-processing strategy are necessary for the harvesting of structural information from the extremely noisy cryo-EM images. The processing pipeline must be flexible and include a wide range of computing tools to be able to handle the large range of possible cryo-EM samples. All steps in the data processing must be validated by clean, unbiased metrics. Here, we present a reference-free pipeline for single-particle cryo-EM structure determination with a strong emphasis on eigenvector data compression, alignment by classification (ABC-4D) and angular reconstitution. All cryo-EM data processing is sensitive to the power-spectrum distribution of the data as a function of spatial frequency and therefore filtering may significantly change the results. The refinement procedures must be carefully monitored as one gradually includes more high-frequency information from the raw data into the analysis.

### 3.1. MSA/classification   

Our most important general tool for the extraction of intrinsic information at all phases of the processing is the MSA eigenvector–eigenvalue data compression and unsupervised classification. These straightforward linear data-reduction techniques allow unprejudiced focusing on the information emerging from the measured data. They also allow the diagnosis of unintended or irrelevant sources of variance in the data, such as unexpected anisotropic magnification (§[Sec sec2.4]2.4), or the diagnosis of ‘false-positive’ particle picking from the background (§[Sec sec2.11]2.11). A low number of eigenimages (∼10–20) may already suffice to, for example, accurately define the Thon ring patterns for CTF defocus determination (§[Sec sec2.5]2.5). Even for large data sets with thousands of raw images, CTF determination (after MSA classification) can therefore be performed within hours on a standard computer.

In the compressed ‘factor space’ spanned by the main eigenvectors of the data set, all distances and similarities are optimized to the variance content of the data set (Borland & van Heel, 1990 ▸). Working in factor space thus makes all processing faster than when operating on full individual images. The latter approach may sometimes require the use of special hardware/software (‘GPU’) to achieve acceptable processing times. Originally, alignments of the molecular images were a prerequisite for applying the MSA un­supervised classification schemes (van Heel, 1984*b* ▸), given the limitation of the computing hardware at the time. Alignments, however, are themselves ‘supervised classification procedures’ which bias the data set. With the rapid increase in computing resources, it became possible to work with much larger data sets and to apply unsupervised MSA classification to images directly prior to their alignment, resulting in grouping of molecular images that happen to lie in the same orientation. Fundamental to this ABC approach is that the decision to group similar images precedes any form of alignment and is thus unbiased (Dube *et al.*, 1993 ▸). One bottleneck associated with large data sets is that a mutual comparison of all molecular images, even in factor space, becomes excessively expensive. A newly implemented ‘hybrid’ unsupervised classification (van Heel *et al.*, 2016 ▸) now allows the grouping of millions of images into thousands of class averages in a matter of hours on standard computers.

A novel aspect of our MSA approaches, especially for final high-resolution refinements, is eigenvector analysis in complex Fourier space (§[Sec sec2.13]2.13). In combination with our MSA ‘modulation’ metric (Borland & van Heel, 1990 ▸), it largely compensates for the over-representation of strong amplitudes (‘squared correlation functions’; van Heel *et al.*, 1992 ▸) and thus helps to focus on the important spatial frequencies. Moreover, spatial frequencies beyond the high-frequency cutoff (of a two-dimensional MSA ring mask) cannot influence the un­supervised classification and thus cannot bias the ABC procedures. The gradual increasing of this cutoff frequency assures that only information that is intrinsically present in the data enters into the refinements, preventing reference bias and overfitting.

A somewhat different form of multivariate statistical analysis has been introduced into cryo-EM in the form of maximum likelihood (ML; Sigworth, 1998 ▸; Scheres *et al.*, 2005 ▸), which has recently had a significant number of successes (Kühlbrandt, 2014 ▸). The PCA approaches (which include MSA) are straightforward variable-reduction techniques that try to describe the variances and correlations in a data set irrespective of why they exist. The ‘exploratory factor analysis’ approaches (which include ML), in contrast, are based on the existence of a correctly formulated underlying causal model. The need for a causal model can be a problem since correlation does not imply causation. We can process the data and find sources of variance differences within the data set by eigenvector analysis and *a posteriori* eliminate irrelevant ones. If a significant contribution in an ML analysis is owing to an unexpected cause (say, an illumination ramp), then this may impede the ML processing because this effect was not part of the causal model.

These two multivariate analysis families are, nevertheless, considered to be similar in the statistical literature (Fabrigar *et al.*, 1999 ▸). A complication in comparing these two multivariate approaches in the cryo-EM literature is that the ML classification procedures in use in cryo-EM have been intimately intertwined with reference-based alignments of two-dimensional images or three-dimensional volumes in a single operation. In the ABC approach, the overall alignment of the data set is entirely separated from the multivariate statistical data compression and the unsupervised classification.

### 3.2. Anisotropic magnification   

A discussion had emerged in the 3DEM forum on the resolution-limiting effect of anisotropic magnification (https://mail.ncmir.ucsd.edu/pipermail/3dem/2014-May/003379.html). Narrow diffraction rings, owing to the presence of small crystals in the sample, must remain rotationally symmetric even in the presence of astigmatism. Astigmatism can cause Thon rings crossing the water rings, yet on average the diffraction power remains concentrated rotationally symmetrically on the same radius. We had found ∼7% magnification anisotropy in earlier data collected using our previously-corrected microscope. A realignment of the previous corrector was performed to remedy this problem. However, when we failed to refine our structure beyond ∼5 Å resolution, we detected a residual 2.6% magnification anisotropy using our MSA spectrum analysis (Fig. 2 ▸). Moreover, we observed different anisotropic magnification parameters in various data sets, implying that these parameters are not instrumental constants. We associate this anisotropy primarily with a residual misalignment of the *C*_S_ corrector, in conjunction with the use of *C*_S_-corrector optics for astigmatism correction. The microscope manufacturer informed us of a revision of the alignment protocols to minimize this anisotropy.

Two papers have since suggested assessing the anisotropy with a test sample to then correct the data by re-interpolation (Grant & Grigorieff, 2015 ▸; Zhao *et al.*, 2015 ▸). In contrast, our anisotropy assessment stems from the collected data and does not require additional measurements. Note that in their case the instrument did not have a *C*_S_ corrector, indicating that the anisotropic magnification issue is not exclusively connected to the use of a *C*_S_ corrector.

In Fig. 2 ▸, the water ring at 2.2 Å is clearly discerned in spite of it being at the Nyquist frequency, where the DQE values drop significantly. We can even locate the ‘super-resolution’ (beyond the Nyquist frequency) water ring at ∼1.8 Å folding back into the spectrum (‘aliasing’). This very low SNR ‘corner’ information is almost never mentioned in publications. It only emerges here owing to the ‘full-data-set’ aspect of the eigenvector analysis, bringing even the weakest of effects to statistical significance. The Falcon II is not a ‘super-resolution’ single-electron detection camera, as is, for example, the Gatan K2 Summit or the Falcon III camera (Kuijper *et al.*, 2015 ▸). Nevertheless, our MSA analysis reveals that significant ‘super-resolution’ information could be recorded.

### 3.3. What is reference-free?   

The reference-bias problem in single-particle analysis was identified early on (Boekema *et al.*, 1986 ▸), and various ‘reference-free’, ‘unbiased’ solutions have since been proposed, including invariant classification (Schatz & van Heel, 1990 ▸) and ABC (Dube *et al.*, 1993 ▸). The reference-free concept has, however, thereafter suffered devaluation. For example, in Penczek *et al.* (1992 ▸) the alignment algorithm is called ‘reference-free’ because the reference images used to start their procedures were chosen at random. Also, ‘two-dimensional classification’ schemes used in connection with maximum-likelihood (ML) approaches (Sigworth, 1998 ▸; Scheres *et al.*, 2005 ▸) are not ‘reference-free’ but use randomly selected starting references. These ML approaches, which use projection matching (PM), are nevertheless often confusingly called ‘reference-free’ (see van Heel, 2013 ▸). PM schemes, per definition, require the alignment of molecular images with many references and are thus prone to reference bias and overfitting. To be fair, much effort with these ML approaches has gone into avoiding reference bias by, for example, removing all high-frequency information from the reference images prior to alignment. In the ABC procedures, in contrast, the orientational parameters of the class members are inherited from the class averages, which in turn have their parameters determined with respect to the full three-dimensional structure by angular reconstitution. The latter procedure is virtually immune to reference bias.

### 3.4. Angular reconstitution *versus* projection matching   

The difference between AR and PM is the source of much misunderstanding. AR is based on finding ‘common projection lines’ (CPLs) between an input image and (just a few) other images, which have already received Euler orientations (van Heel, 1987 ▸; van Heel *et al.*, 2012 ▸). These other images can be class averages (during the startup phase) or an ‘anchor set’ derived from a three-dimensional reconstruction (Schatz *et al.*, 1995 ▸). The CPL theorem (van Heel, 1987 ▸; van Heel *et al.*, 2012 ▸) is the real-space equivalent of the Fourier-space ‘central slice’ or ‘common-line’ theorem (De Rosier & Klug, 1968 ▸; Sigworth, 2015 ▸; van Heel & Harauz, 1986 ▸; Crowther, 1971 ▸; Bracewell, 1956 ▸). Projection matching (van Heel, 1984*a* ▸; Penczek *et al.*, 1994 ▸), in contrast, requires a multi-reference alignment (van Heel & Stöffler-Meilicke, 1985 ▸) of the input image with respect to all possible projection images of a single three-dimensional reconstruction (van Heel *et al.*, 2000 ▸; Grigorieff, 2007 ▸; Bai *et al.*, 2015 ▸; Sigworth, 2015 ▸) or multiple three-dimensional reconstructions (Klaholz *et al.*, 2004 ▸; Zhou *et al.*, 2015 ▸), representing a large computational effort.

In the AR approach we only use a low number of projections to assign Euler angles to an image. A low number *n* of noise-free projections fully defines a three-dimensional object to high resolution (De Rosier & Klug, 1968 ▸; Sigworth, 2015 ▸; van Heel & Harauz, 1986 ▸; Crowther, 1971 ▸). To achieve a three-dimensional reconstruction with a uniform 3 Å resolution (*d* = 3 Å) for a 300 Å diameter oligomer (*D* = 300 Å) with *D*6 symmetry (*N*_sym_ = 12), one only needs (*n* ≃ 20) noise-free projections (*d* ≤ 2*D*/*n* *N*_sym_). This three-dimensional reconstruction, in turn, defines the thousands of possible two-dimensional projections used for alignments in PM. For a full 1° coverage of the asymmetric triangle of a *D*6 symmetric structure ∼3438 PM references are required (∼41 253/12). In AR, in contrast, the Euler angles are derived directly from those ∼20 projections, avoiding those costly alignments. AR thus exploits the same information as PM. Although there can be differences in implementation, AR and PM refinements are thus in principle equivalent. However, since AR refinements focus on only the core three-dimensional information, they are (much) faster.

The AR–PM equivalence applies only to the refinement phase of the processing, once the three-dimensional Fourier space has been uniformly filled with information. In the early phases of processing we only have a few class averages, that is a few clearly defined central sections in an otherwise empty Fourier space. Historically, PM was introduced in conjunction with random Euler-angle assignments for starting up three-dimensional reconstructions (van Heel, 1984*a* ▸; van Heel & Harauz, 1986 ▸). However, PM was found to often land in local minima owing to missing three-dimensional information (wedges in Fourier space; van Heel, 1984*a* ▸; Harauz & Ottensmeyer, 1984 ▸; Sanz-García *et al.*, 2010 ▸; van Heel *et al.*, 2000 ▸). Our developments then moved to AR (van Heel, 1987 ▸), which is robustly based on measured data alone, *i.e.* on the available central sections, ignoring the missing wedges. The effectiveness of AR is demonstrated by our routine of starting up Euler-angle assignments at random (§[Sec sec2.10]2.10). Note that ‘starting models’ for PM-based refinement procedures in packages such as *FREALIGN* (Grigorieff, 2007 ▸) and *RELION* (Scheres, 2012 ▸) are often generated by AR or its various recent derivatives (Sigworth, 2015 ▸; Cheng *et al.*, 2015 ▸). We conclude that the culture of using external starting models is owing to the popularity of PM refinement procedures, causing an unnecessary methodological rift between the early and the later phases of the analysis.

### 3.5. Anisotropic particle distributions lead to anisotropic resolution   

In our sample many more ‘top views’ than ‘side views’ were present, generating better statistics in the preferred orientation. We thus introduced two restricted FSC variants, FSC_β<60°_ and FSC_β>60°_, to assess this effect (Fig. 11 ▸). The β angle used is strictly speaking the shortest angular distance to the main preferred orientation, but it coincides with the β Euler angle in the case of a *D*6 symmetric structure. Restricted FSCs have been used in tomography to account for tilt limitations (Diebolder *et al.*, 2015 ▸). Preferred particle orientations have been argued to cause extensions of the three-dimensional reconstructions (Boisset *et al.*, 1998 ▸). Our linear reconstructions using the ‘exact-filter’ weighted back-projection algorithm (Harauz & van Heel, 1986 ▸) do not show such extensions, indicating that these distortions were rather artefacts of the iterative and nonlinear *SIRT* algorithm used (the overabundant views receive a disproportional weight during the *SIRT* iterations).

### 3.6. Overall quality of our global map   

Our *L. terrestris* haemoglobin reconstruction (global FSC resolution of ∼3.8 Å) is comparable in resolution to X-ray crystallography maps (Royer *et al.*, 2006 ▸; Ruggiero Bachega *et al.*, 2015 ▸). In the best parts of our map the resolution is sufficient to distinguish between large side chains. The resolution achieved is also sufficient to elucidate the environment of the haem groups, with densities for the proximal and distal histidines (Fig. 12 ▸ *d*). Some acidic residues have apparently lost side-chain density, which is likely to be a consequence of radiation damage (see Bartesaghi *et al.*, 2014 ▸; Allegretti *et al.*, 2014 ▸). The most stable part of the map is the linker-chain core of the complex; the haem-containing globins on the outer periphery have a lower resolution owing to flexibility/movement. In X-ray studies of crystals containing the entire complex in the asymmetric unit the situation is reversed: the inner parts of the L1/L2/L3 triple coiled-coil helix are not well resolved owing to local flexibility (Royer *et al.*, 2000 ▸, 2006 ▸) compared with the outer domains involved in crystal contacts.

As a rule of thumb, the resolution achievable in X-ray crystallography is a function of the size of the crystallographic asymmetric unit (assuming everything else to be comparable). The smaller the unit cell, the more constrained the molecules are. Thus, a 2.6 Å resolution structure could be achieved from a crystal with only part of the 1/12th subunit in the asymmetric unit (a trimer of *abcd* tetramers; Strand *et al.*, 2004 ▸). The *G. paulistusa* X-ray crystal structure at 3.2 Å resolution (Ruggiero Bachega *et al.*, 2015 ▸) was based on crystals containing one quarter of the complex (three protomers) in the asymmetric unit. The X-ray crystal structure of *L. terrestris* haemoglobin contains two full *D*6 molecules in the asymmetric unit (24 protomers) and was first resolved to 5.5 Å resolution (Royer *et al.*, 2000 ▸); it was subsequently refined to 3.5 Å resolution by merging information from different crystal forms (Royer *et al.*, 2006 ▸). We conclude that our cryo-EM resolution is in line with the size-related resolutions achieved in X-ray crystallography, and is also primarily limited by the size and flexibilities of the complex.

In X-ray crystallography this limitation is fundamental in that all variations average out during data collection. In single-particle cryo-EM, however, each molecule remains accessible as an independent measurement for future refinement. This is equally true for any fraction of the molecule; one can thus *a posteriori* refine separate parts of the complex in cryo-EM. This type of local refinement compares with having only a part of the complex in the asymmetric unit in crystallography. The study of smaller individual biological complexes (not necessarily part of a larger complex) remains an important motivation for methodology developments. The ABC-4D approach facilitates the study of smaller complexes by avoiding the ‘Einstein in the noise’ pitfall associated with correlation alignments (Henderson, 2013 ▸; Schatz, 1992 ▸).

The wealth of structural information currently available on (mainly tetrameric) haemoglobins is almost exclusively the result of X-ray crystallography experiments. Crystallization conditions and crystal bonds, however, may impose conformational states on the complex that are independent of the actual functional state of the haemoglobin. The detailed mechanism of oxygen binding and the associated conformational state changes are thus still not fully understood. Single-particle cryo-EM with its potential to *a posteriori* focus on small details may provide new insights into these open questions.

## 4. Conclusions   

The introduction of direct electron detectors, in combination with decades of developments in instrumentation and data analysis, has led to a ‘resolution revolution’ in single-particle cryo-EM. The processing of cryo-EM data, however, still largely follows slow projection-matching approaches, which are prone to reference bias. We show that angular reconstitution is a universal and fast approach for Euler-angle determination, including during structural refinement. Moreover, dealing with the image information in a compressed eigenvector space is inherently much more efficient than dealing with the raw image information directly. We have demonstrated the potential of ABC-4D by solving the structure of *L. terrestris* haemoglobin to near-atomic resolution. The quasi-atomic environment of a haem group has been elucidated by single-particle cryo-EM for the first time. The preferred orientations of the particles lead to different reproducible resolutions (FSC) in different orientations. FSC_β<60°_ and FSC_β>60°_ are straightforward metrics for assessing this effect. Our ABC-4D processing pipeline is virtually reference-free and can thus be expected to work for smaller biological structures than have hitherto been considered feasible.

## Supplementary Material

Supplementary Movie S1. A movie that highlights how the protomer fits in the whole molecule.. DOI: 10.1107/S2052252517010922/kf5002sup1.mp4

PDB reference: *Lumbricus terrestris* haemoglobin, 5m3l

## Figures and Tables

**Figure 13:**
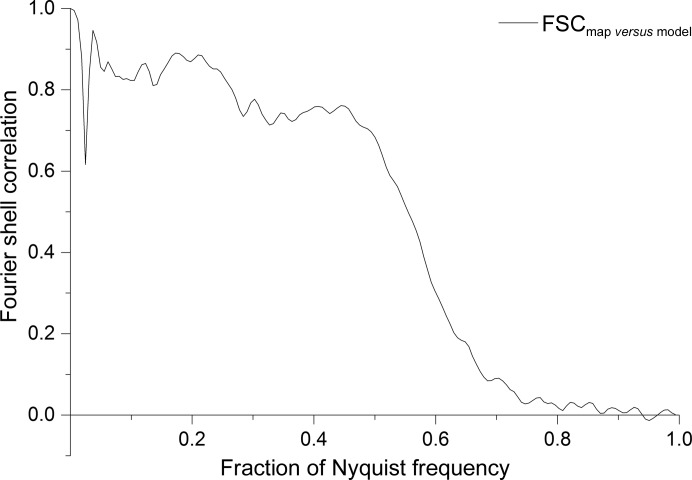
Fourier shell correlation plot of the model (protomer) *versus* the map (masked 1/12th subunit; van Heel *et al.*, 2000 ▸). The atomic model was deposited as PDB entry 5m3l.

**Table 1:** Model-refinement statistics

All-atom contacts	
*MolProbity* clashscore	9.98 (97th percentile, *N* = 37, >3 Å)
Protein geometry	
Poor rotamers (%)	0.59
Favoured rotamers (%)	94.88
Ramachandran plot	
Favoured (%)	91.46
Allowed (%)	7.40
Outliers (%)	1.14
*MolProbity* score	2.02 (100th percentile, *N* = 342, 3.25–4.05 Å)
C^β^ deviations > 0.25 Å (%)	0.31
Bad bonds (%)	0.01
Bad angles (%)	0.08
R.m.s. deviations (refined atoms)	
Bond lengths (Å)	0.009
Bond angles (°)	1.023
Average FSC	0.81
*R*_free_	0.34

## Appendix A Materials and methods

### A1. Sample preparation

Blood of the earthworm *L. terrestris* was extracted from the seventh segment of the body of some 20 individual worms and the (pooled) blood was diluted 30 times with 0.1 *M* Tris–HCl pH 7.0, 1 m*M* EDTA. The diluted sample (3 µl) was applied onto a Quantifoil grid (R2/2, Quantifoil Micro Tools GmbH) in a Vitrobot Mark IV (FEI) at 22°C and 100% humidity and, after a 2.5 s blotting time, was plunge-frozen into liquid ethane.

### A2. EM data collection

The micrographs were recorded using the *EPU* software under low-dose conditions on a Titan Krios microscope (FEI, NeCEN) equipped with a 300 keV XFEG and a *C*_S_ corrector. The data were recorded using a 4096 × 4096 pixel Falcon II camera at a magnification of 59 000× (pixel size of 1.12 Å) at a defocus range of 1–1.2 µm underfocus. Data acquisition was performed under the control of the *EPU* software (FEI) in movie mode, collecting series of seven frames per movie (5235 movies in total) with a total electron exposure of ∼40 e^−^ Å^−2^. The average pixel size, originally calibrated at 1.12 Å per pixel, was later refined to 1.11 Å per pixel as a result of the correction for anisotropic magnification by re-interpolation (for details, see §[Sec sec2.4]2.4).

### A3. Cryo-EM image processing

All image processing was performed in the context of the *IMAGIC-*4*D* software package (van Heel *et al.*, 1996 ▸, 2012 ▸). The CPU-intensive tasks such as particle picking and MSA eigenvector data compression were performed on MPI clusters running Linux, typically using 32–128 cores. Interactive test runs, general pre-processing, classifications, final three-dimensional/four-dimensional reconstructions *etc.* were typically performed on desktop/notebook computers under Windows 10 or Linux/Ubuntu operating systems.

### A4. Model building and refinement

To build the atomic model of *L. terrestris* haemoglobin, an existing X-ray model (PDB entry 2gtl; Royer *et al.*, 2006 ▸) was initially fitted manually into the electron density of one of the protomers of the final cryo-EM map with *UCSF Chimera* (Pettersen *et al.*, 2004 ▸). This starting model had an initial *R*_free_ factor of 48% and was iteratively refined using *REFMAC*5 (Murshudov *et al.*, 2011 ▸) and manual model building with *Coot* (Emsley *et al.*, 2010 ▸). In the final stages, real-space and reciprocal-space refinement were also performed with *PHENIX* (Adams *et al.*, 2010 ▸), leading to a final *R*_free_ factor of 34%. The final model was checked for geometry, close contacts and bond parameters using *MolProbity* (Chen *et al.*, 2010 ▸; Davis *et al.*, 2007 ▸). Refinement and model-building statistics are provided in Table 1 ▸; the FSC of model (protomer) *versus* map is shown in Fig. 13 ▸. Graphic representations of the fitted coordinates into electron density and the final cryo-EM map were produced using the *PyMOL* (v.1.8; Schrödinger) and *UCSF Chimera* (Pettersen *et al.*, 2004 ▸) packages.
